# Bestatin inhibits the development of cutaneous melanoma by inhibiting the expression of LTA4H

**DOI:** 10.3389/fimmu.2026.1901844

**Published:** 2026-07-28

**Authors:** Pan Luo, Mingyi Yang, Yani Su, Pengfei Wen, Xiqin Lu

**Affiliations:** 1 Department of Plastic Surgery, Beijing Chaoyang Hospital Affiliated to Capital Medical University, Beijing, China; 2 Department of Joint Surgery, HongHui Hospital, Xi’an Jiaotong University, Xi’an, China; 3 Department of Radiotherapy, Tangdu Hospital, The Fourth Military Medical University, Xi’an, Shaanxi, China; 4 Department of Hand Surgery, Honghui Hospital, Xi’an Jiaotong University, Xi’an, China

**Keywords:** cutaneous melanoma, drug targets, gene therapy, genetics, Mendelian randomization

## Abstract

**Objective:**

In this study, we used Mendelian randomization analysis to explore the causal relationships between drug targets and cutaneous melanoma (CM) and subsequently screened for new drug targets for CM. In addition, we verified whether targeted drugs could inhibit the development of CM in CM cells by suppressing the expression of target genes.

**Methods:**

The eQTL data of 2,664 drug target genes (exposures) were sourced from the eQTLGen database. The GWAS summary data for CM (outcome) were obtained from the UK Biobank and FinnGen databases. The inverse-variance weighted (IVW) model was used as the primary analytical method. For the cell experiments, we first conducted RT-PCR and western blot analyses in three CM cell lines to validate the expression of LTA4H. LTA4H was subsequently knocked down and overexpressed in CM cell lines, and the proliferation, migration, invasion, and apoptosis of CM cells were evaluated. Finally, bestatin was used to stimulate CM cells to examine its effect on their malignant functions.

**Results:**

MR, GEO2R, and PheWAS analyses revealed that high expression of LTA4H can promote the development of CM. In cell experiments, compared with normal fibroblasts, melanoma cells highly expressed LTA4H. Moreover, after LTA4H was knocked down in SK-MEL-28 cells, their malignant functions decreased. After LTA4H was overexpressed in A-375 cells, the degree of malignancy increased. Finally, bestatin inhibited the malignancy of A-375 cells through the regulation of LTA4H.

**Conclusion:**

In this study, MR analysis revealed that high expression of LTA4H can promote the occurrence of CM. In addition, the results of the cell experiments revealed that bestatin can inhibit the development of melanoma cells by reducing the expression of LTA4H.

## Highlights

- First identification and validation of LTA4H as a novel tumour-promoting target in cutaneous melanoma.
- A rigorous study design enhances the robustness of the conclusions through multistep, multidata source cross-validation.
- Prospective assessment of potential side effects of targeted therapy and identification of the core target.
- Validation of drug repurposing potential at the cellular level, accelerating potential clinical translation.

## 1 Introduction

Cutaneous melanoma (CM) is a life-threatening type of skin cancer (1). Although CM accounts for only 4% of all occurrences of skin cancer, it leads to 75% of fatalities from skin cancer (2–4). The aetiology of CM often involves environmental and genetic factors (5). Ultraviolet radiation (UVR) and low melanin levels are major risk factors for CM (6). Despite advances in early detection and treatment, CM remains challenging to manage (7). Therefore, identifying novel drug targets is essential for improving prevention and therapeutic outcomes.

Single nucleotide polymorphisms (SNPs) linked to illnesses identified via genome-wide association studies (GWASs) represent an underappreciated source of random human data that can assist in identifying and validating pharmacological targets (8). Genetic variations are used as instrumental variables (IVs) in Mendelian randomization (MR), a potent statistical method for identifying and assessing causal relationships between exposures and outcomes (9, 10). Furthermore, the utilization of publicly accessible data from two independent, large-scale GWASs enables the design of more reliable MR analyses. In the specific field of drug target MR analysis, researchers frequently employ IVs that are cis-expression quantitative trait loci (cis-eQTLs) found close to the genome of medication target genes (11). These cis-eQTLs can regulate the expression levels of their associated genes. By using MR in this manner, researchers can gain insights into the causal effects of gene expression on disease susceptibility (12, 13). This approach has been effectively used to investigate several illnesses, including aortic aneurysms, inflammatory bowel disease, and multiple sclerosis (14–16).

In this study, cis-eQTLs associated with drug targets were used as exposure indicators, and summary data from CM GWASs were used as outcome indicators to conduct MR analysis to explore whether drug targets have a direct effect on CM. We conducted a Mendelian randomization analysis, which was followed by screening new drug targets for CM. In addition, we verified whether the screened target genes could promote the development of melanoma in CM cells and whether relevant targeted drugs could inhibit the development of CM.

## 2 Methods

### 2.1 Identification of cis-eQTLs linked to drug target genes

We obtained eQTL data from the eQTLGen Consortium (https://eqtlgen.org/), which included 31,684 cis-eQTLs and 16,987 genes from blood biopsies. The majority of the participants had European ancestry. More detailed information regarding the characteristics of the participants is presented in the original work (17). The list of druggable genes utilized in this study was derived from a previous study that defined proteins with draggability potential by combining data from many GWASs using computational techniques. This earlier work associated proteins with existing drugs and yielded a list of 4,463 druggable genes (11). Because gene eQTLs are more likely to regulate gene expression near the gene of interest, especially in drug development scenarios, we selected only SNPs within 5 kb upstream and downstream of each druggable gene. Finally, we were able to identify 2,664 drug target genes associated with eQTLs.

### 2.2 Outcome data

During the initial phase of the analysis, GWAS summary data on CM were obtained from the UK Biobank. Specifically, this dataset encompassed a total of 375,767 individuals, among whom 37,510 were diagnosed with CM and 372,016 served as part of the control group. During the replication analysis phase, GWAS summary data for CM were sourced from the FinnGen R11 biobank. The replication cohort for this study comprised 3,932 CM patients and 244,907 individuals in the control group. The summary statistics are made available by FinnGen. Data from the FinnGen project are publicly available and can be retrieved through their official site at https://finngen.gitbook.io/documentation.

### 2.3 MR analysis

Two-sample MR analysis was performed to assess potential causal relationships between specific drug target genes and susceptibility to CM. Independently significant SNPs (P < 5 × 10^–8^) were selected for each drug target gene using the “clump” function in PLINK 1.9, with an r² cut-off of 0.001 and a clumping window of 10,000 kb (18). SNPs that passed this filtering were used as instrumental variables (IVs); those with F-statistics < 10 were excluded as weak instruments.

The inverse-variance weighted (IVW) method was applied for primary causal inference. Horizontal pleiotropy was evaluated using the MR-Egger intercept test, and heterogeneity among IVs was assessed with Cochran’s Q statistic (P < 0.05 indicated significant heterogeneity). Outliers were detected and corrected via the MR-PRESSO global test.

To account for multiple testing, a false discovery rate (FDR) correction was applied. In the discovery cohort, associations with an FDR-adjusted P < 0.05 were considered significant; in the replication phase, associations with an IVW P < 0.05 were retained for further evaluation.

We carried out all analyses in R (version 4.2.0) using the TwoSampleMR and MRPRESSO packages, which are available at www.r-project.org.

### 2.4 Screening of differentially expressed genes associated with CM

The publicly accessible GEO database (https://www.ncbi.nlm.nih.gov/geo/), which specializes in generating a vast array of functional genomic data, was used as a source of gene expression data (19). Within this repository, we downloaded a dataset known as GSE46517, which pertains to a chip expression profile. This particular dataset included a total of 31 primary CM samples and an additional 7 samples derived from normal skin tissue (20). The GEO2R tool (https://www.ncbi.nlm.nih.gov/geo/geo2r/) functions as an integrated feature of the GEO database for comparative data analysis. The use of GEO2R allowed us to conduct a thorough analysis aimed at identifying DEGs that are present between CM samples and healthy control samples. We subsequently searched for melanin-related drug target genes among the DEGs to determine whether these genes exhibited differential expression at the RNA level in CM patients.

### 2.5 PheWAS analysis

With information sourced from the PheWeb database (https://pheweb.org/), we conducted a phenome-wide association study (PheWAS) to investigate both the therapeutic and unintended effects of the proposed targets (21). Approximately 1,500 continuous phenotypes were utilized in the original study. The UK Biobank provided the individuals in the exome sequencing subgroup. A comprehensive explanation of the methods was included in the primary source (22). Understanding the genetic foundation of complex characteristics and evaluating the safety and effectiveness of pharmacological targets are facilitated by this thorough PheWAS.

### 2.6 Cell culture and transfection

A-375 cells (IM-H332; Xiamen Immocell Biotechnology, China) were cultured in DMEM supplemented with 10% fetal bovine serum (FBS) and 1% penicillin/streptomycin (P/S). HSF cells (IM-H032; Xiamen Immocell Biotechnology, China) were cultured in DMEM supplemented with 10% FBS, 1% P/S, 0.005 mg/ml insulin, 5 ng/ml basic fibroblast growth factor (bFGF), 1 μg/ml hydrocortisone, 50 μg/ml ascorbic acid (vitamin C), and 7.5 mM L-glutamine. SK-MEL-28 cells (IM-H463; Xiamen Immocell Biotechnology, China) were cultured in MEM supplemented with 10% yeast folate-modified FBS (YMFBS) and 1% P/S. M14 cells (IM-H174; Xiamen Immocell Biotechnology, China) were cultured in DMEM supplemented with 10% FBS and 1% P/S. These four cell lines were housed in a humidified environment at 37 °C and infused with 5% CO_2_. Bestatin (HY-B0134; MedChemExpress LLC, USA) was obtained from MCE. For plasmid transfection, jetPRIME^®^ Transfection Reagent (101000046; Polyplus-transfection SA, France) was used.

### 2.7 CCK8 assay

In 96-well plates (3599, Corning, USA), SK-MEL-28 and A-375 cells were inoculated at a density of 2 × 10³ cells per well. Upon adherence, each well received 110 μl of a concoction of CCK8 stock solution and serum-free medium, which composed the working solution. The samples were incubated in a 37 °C incubator for 1.5 h, after which the absorbance at 450 nm was quantified via a microplate reader (Varioskan LUX, Thermo Fisher Scientific, USA). The RNA pellets were reconstituted in 12 μL of RNase-free water.

### 2.8 RT-PCR

The cell samples, which were immersed in 1 ml of TRIzol reagent and subjected to intense vertexing for 15 seconds, were incubated at room temperature for 5 min. The samples were subsequently centrifuged at 12,000 rpm for 15 min at 4 °C. After the supernatant was removed, the samples received 500 μl of isopropanol and were inverted, followed by a 10 min incubation at room temperature. Another centrifugation at 12,000 rpm for 10 min at 4 °C preceded the removal of the supernatant. Next, 75% ethanol, prepared in RNase-free ddH_2_O and chilled in a 4 °C refrigerator, was added to EP tubes in a volume of 1 ml and mixed by gentle inversion. Subsequent centrifugation at 12,000 rpm for 15 min at 4 °C resulted in the discarding of the supernatant and the assembly being left at room temperature for 5 min. The RNA concentration was gauged using a microvolume UV-Vis spectrophotometer (Biospectrometer basic, Eppendorf SE, Germany). The reverse transcription process was performed using a reverse transcription kit (KR118-02; TIANGEN BIOTECH, China), culminating in RT-qPCR analysis with SuperReal PreMix reagent (FP205-02; TIANGEN BIOTECH, China) on a StepOnePlus Real-Time PCR System (4376600; Thermo Fisher Scientific, USA), with GAPDH serving as the internal control. The primer sequences are detailed in **Supplementary Table 1**, and gene expression levels were determined using the 2^(−ΔΔCt)^ method.

### 2.9 EdU assay

After being cultured at a concentration of 2 × 10^4^ cells/mL in 6-well plates, SK-MEL-28 and A-375 cells were subjected to EdU cell proliferation detection using an EdU cell proliferation assay kit (C0078S; Beyotime Biotechnology, China). Prewarmed EdU working solution (20 μM) was added at equal concentrations. After 2 hours of incubation at 37 °C, 1 ml of 4% polyformaldehyde (P1110; Beijing Solarbio Science & Technology, China) was added to fix the cells for 15 min at room temperature. Once the fixative was discarded, the cells were rinsed with wash buffer, and 1 ml of Triton permeabilization solution (T6200G; Beijing Bio-Top Technology, China) was added, followed by rinsing. Each well then received 0.5 ml of prepared Click Additive Solution and was incubated in the dark for 30 min at room temperature, followed by rinsing. DAPI staining was applied, and the cells were scrutinized and imaged under a fluorescence microscope (Ti2, Nikon Corporation, Japan). The cell proliferation rate (%) was computed as the proportion of EdU-positive cells to DAPI-positive cells.

### 2.10 Wound healing assay

After transfection, SK-MEL-28 and A-375 cells were seeded in 6-well plates and allowed to reach 90% confluence. A simulated wound was generated in a cell monolayer with a 200 μL pipette tip, and the cells were cultured in medium supplemented with 1% FBS for 24 and 48 h. Microscopy images were captured at 0 h, 24 h, and 48 h (XD-202; Nanjing Jiangnan Novel Optics, China).

### 2.11 Transwell migration assay

Migration assays with SK-MEL-28 and A-375 cells at a density of 2 × 10^5^ cells/well were performed using Transwell chambers (354480; Corning, USA). After being suspended in serum-free medium, the cells were placed in the upper chambers, while the lower chambers contained medium enriched with FBS. Following 24 and 48 h of incubation, the cells that migrated through the membrane were rinsed with PBS, fixed with 4% polyformaldehyde (P1110; Beijing Solarbio Science & Technology, China) for 30 min, and stained with 0.1% crystal violet (G1063; Beijing Solarbio Science & Technology, China) for 30 min. The stained chambers were then washed twice with distilled water; any excess cells were removed with a cotton swab, and the chambers were inverted and dried on absorbent paper, which was subsequently marked externally. The cells were observed and counted with an inverted microscope (XD-202; Nanjing Jiangnan Novel Optics, China).

### 2.12 Transwell invasion assay

Invasion assays commenced with the addition of 100–200 μL of diluted Matrigel (M8370; Beijing Solarbio Science & Technology, China) to the central region of the upper chamber in the Transwell inserts, followed by incubation at 37 °C for 5 h to form a gel. SK-MEL-28 and A-375 cells, at a density of 2 × 10^5^ cells/well, were then suspended in 200 μL of serum-free medium and introduced into the upper chambers, with FBS-enriched medium in the lower chambers. After 24 and 48 h of incubation, the invading cells were rinsed with PBS, fixed with 4% polyformaldehyde (P1110; Beijing Solarbio Science & Technology, China) for 30 min, and stained with 0.1% crystal violet (G1063; Beijing Solarbio Science & Technology, China) for 30 min. The cells were subsequently washed twice with distilled water, after which the residual cells were removed with a cotton swab. Dried chambers, placed upside down on absorbent paper and marked externally, were examined and counted under an inverted microscope (XD-202, Nanjing Jiangnan Novel Optics, China).

### 2.13 Western blot

Upon the addition of lysis buffer to the cells, protease and phosphatase inhibitors (CW2383; Jiangsu Cowin Biotech, China) were introduced, facilitating the complete lysis and extraction of total proteins. The protein concentration was determined using a protein assay kit (23227; Thermo Fisher Scientific, USA). The samples were subjected to SDS-PAGE, after which the proteins were transferred to a PVDF membrane (IPVH00010; Merck KGaA; Darmstadt, Germany). The blocking step was achieved with 5% fat-free milk (A600669-0250; Sangon Biotech, China) by shaking for an hour at room temperature. Primary antibodies, diluted to a concentration of 3% BSA (A6020; Beijing Bio-Top Technology, China), were incubated overnight at 4 °C in a hybridization bag. After three washes with TBST, the cells were incubated with secondary antibodies diluted in 3% BSA for an hour. The samples were subsequently incubated in developing solution (MA0186-2; Dalian Meilun Biotechnology, China), after which images were captured on a multifunctional imaging workstation (GelView 6000Pro II; Guangzhou Biolux Bio-Tech Co., Ltd., China). Semiquantitative analysis was performed using ImageJ software. The primary antibodies used included LTA4H (MA5-34875; Thermo Fisher Scientific, USA) and Flag (ab205606; Abcam, UK).

### 2.14 Clonogenic assay

After being seeded in 6-well plates (80346-1510; Citotest Labware Manufacturing, China) pretreated with 0.1% gelatine (C0316; Beyotime Biotechnology, China), SK-MEL-28 and A-375 cells were allowed to establish themselves. They were then nurtured in a humidified atmosphere at 37 °C and 5% CO_2_ for 14 days. The plates were retrieved, washed twice with PBS, and fixed with 1 ml of 4% polyformaldehyde (P1110; Beijing Solarbio Science & Technology, China) for 15 min. After 15 min of staining with 0.1% crystal violet (G1063; Beijing Solarbio Science & Technology, China), the plates were rinsed with tap water to remove surplus stain before drying. Once dried, the plates were imaged under a microscope (XD-202; Nanjing Jiangnan Novel Optics, China), and the colony count was analyzed using ImageJ software.

### 2.15 TUNEL staining

Cells grown on culture inserts (80346-1510; Citotest Labware Manufacturing, China), including SK-MEL-28 and A-375, were incubated in PBS and fixed with 4% polyformaldehyde (P1110; Beijing Solarbio Science & Technology, China) for 30 min. The cells were then washed with PBS and permeabilized with 0.3% Triton X-100 (T6200G; Beijing Bio-Top Technology, China) for 5 min at room temperature. Each insert then received 50 μL of TUNEL reaction mixture (C1090; Beyotime Biotechnology, China) and was incubated in the dark for 60 min at 37 °C. After three PBS washes, the cells were observed and imaged under a fluorescence microscope (Ti2, Nikon Corporation, Japan), with the excitation wavelength set at 550 nm and the emission wavelength set at 570 nm, reflecting red fluorescence.

### 2.16 JC-1 staining

Following transfection or drug treatment, SK-MEL-28 and A-375 cells were washed with PBS and stained with 1 ml of JC-1 staining working solution (C2003; Beyotime Biotechnology, China). After thorough blending, the mixture was incubated at 37 °C for 20 min. The supernatant was extracted, and the cells were rinsed twice with JC-1 staining buffer. Two milliliters of cell culture medium was then added, and confocal images were captured using a laser scanning confocal microscope (DM3000 LED, Leica Microsystems GmbH, Germany).

### 2.17 Statistical analysis

Each sample was subjected to the experiment three times, and the results are shown as the means ± SDs. GraphPad 8.0 was used for all computations. Additionally, one-way ANOVA with Tukey’s *post hoc* test was used to compare more than two groups. An independent samples t-test was used to compare two groups. Differences with P < 0.05 were considered to be statistically significant.

## 3 Results

### 3.1 Study design

**Figure 1:**
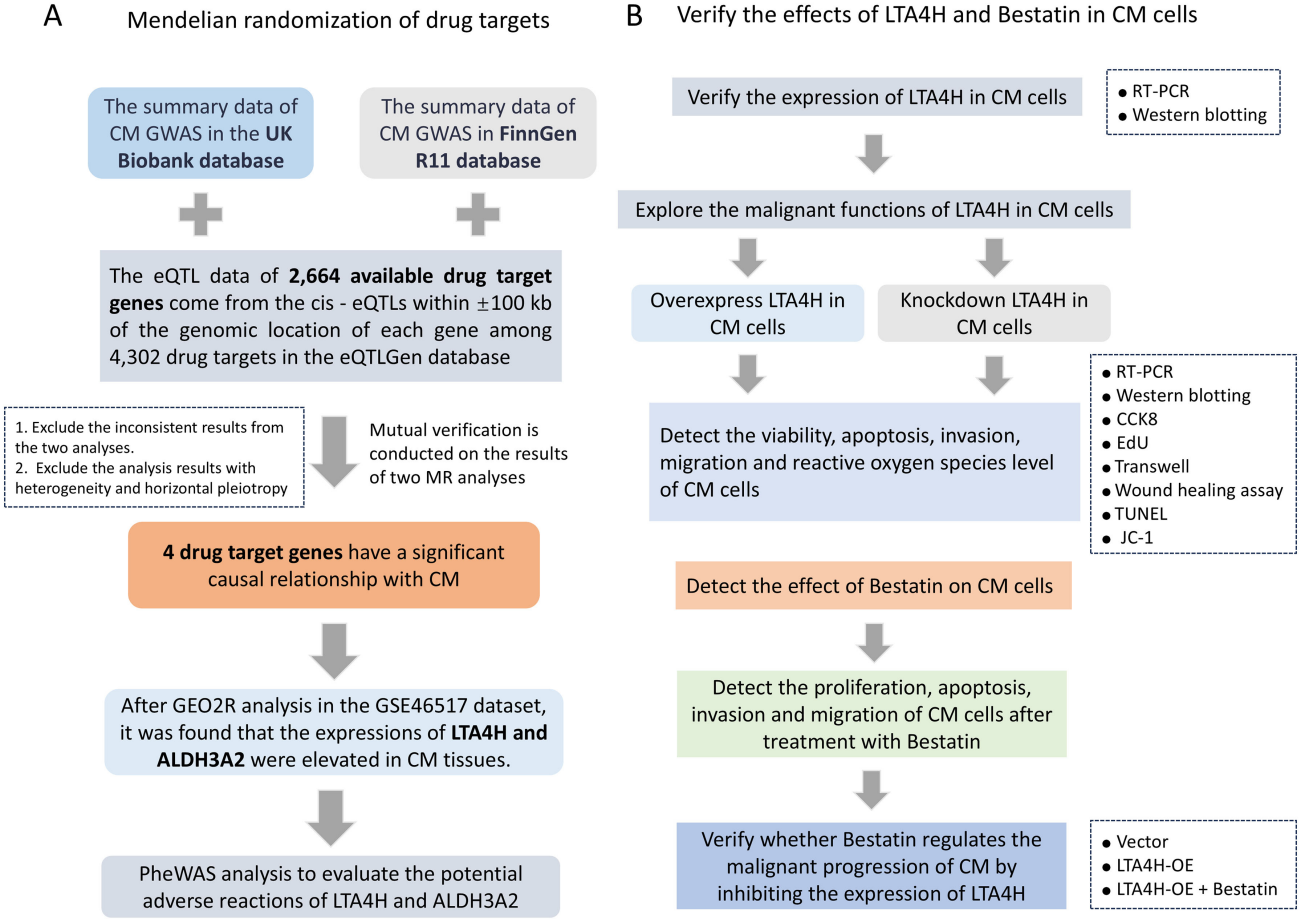
Flowchart of the study. **(A)**​ Screening of drug target genes using public databases. **(B)**​ Validation process in cellular experiments.

The study workflow is shown in **Figure 1**. In the discovery phase, cis-eQTL data from the eQTLGen Consortium were used to identify 2,644 drug target genes, followed by MR analysis using pooled CM patient data from the UK Biobank. In the replication phase, CM GWAS summary data from FinnGen R11 were used as outcomes. These two MR analyses revealed four CM-associated drug target genes.

Differentially expressed genes (DEGs) were subsequently screened to identify melanin-related drug target genes and validate their RNA-level dysregulation in CM. A phenome-wide association study (PheWAS) was performed to assess the potential side effects of targeting LTA4H and ALDH3A2. Because there was no clear association between ALDH3A2 and CM in the presence of PheWAS, subsequent cellular experiments focused on LTA4H.

The cellular experiments included (1) detecting LTA4H expression at the mRNA and protein levels in CM cells (2), investigating the role of LTA4H in malignancy via knockdown and overexpression, and (3) determining whether bestatin regulates CM progression by inhibiting LTA4H.

### 3.2 MR analysis

**Figure 2:**
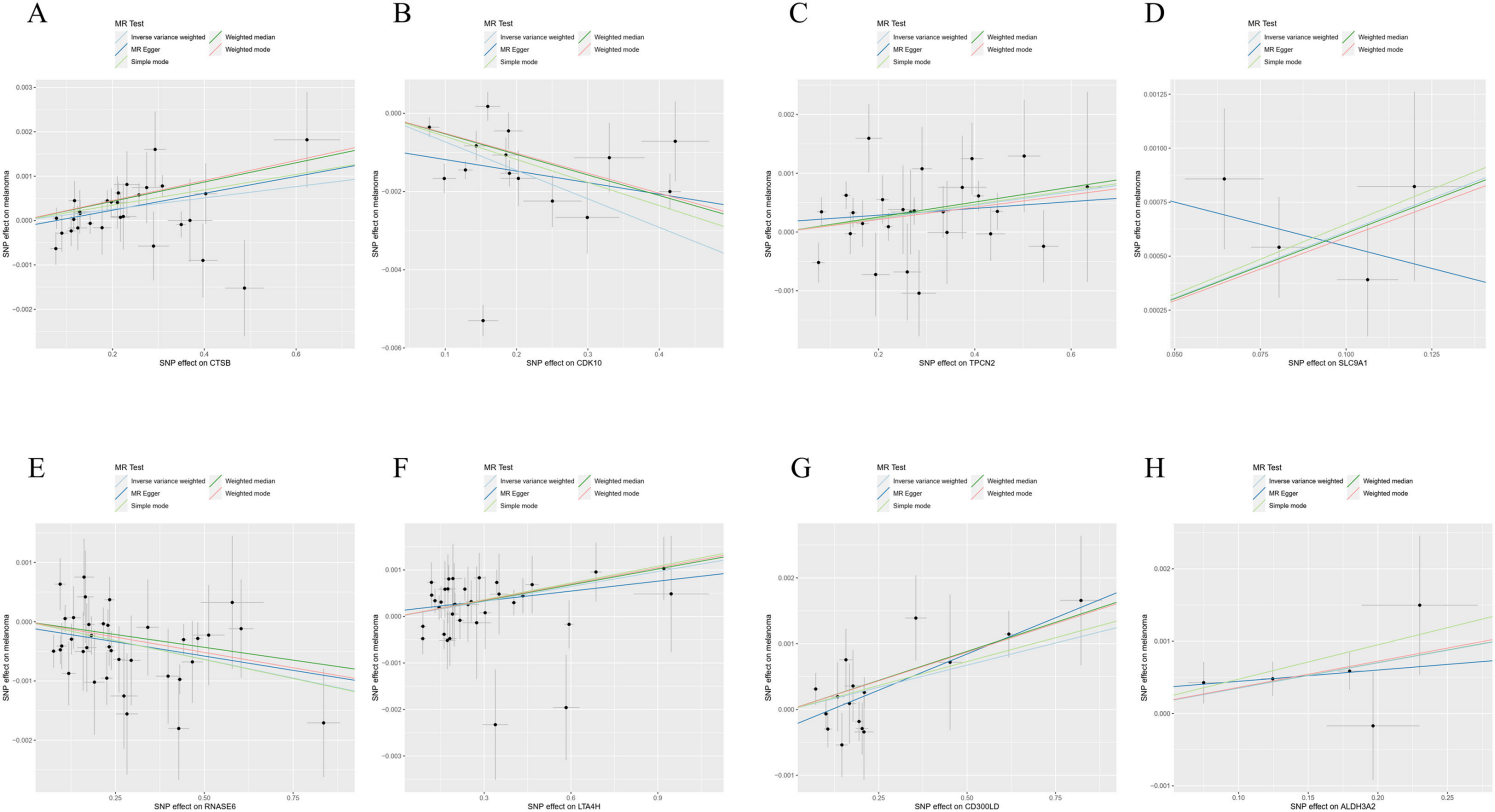
Scatter plot of Mendel randomization results. **(A)**, CTSB; **(B)**, CDK10; **(C)**, TPCN2; **(D)**, SLC9A1; **(E)**, RNASE6; **(F)**, LTA4H; **(G)**, CD300LD; **(H)**, ALDH3A2.

In the discovery and replication analyses, IVW revealed a statistically significant causal relationship (P-FDR < 0.05) between eight drug target genes and CM, including CTSB, CDK10, TPCN2, SLC9A1, RNASE6, LTA4H, CD300LD, and ALDH3A2 (**Table 1**, **Figures 2A–H**). IV information related to the eight drug target genes is shown in **Supplementary Table 2**. **Supplementary Tables 3** and **S4** show the results of the two MR analyses. Among them, increased expression of CTSB, TPCN2, SLC9A1, LTA4H, CD300LD, and ALDH3A2 increased the risk of CM (P-FDR < 0.05, beta > 0). Reduced expression of RNASE6 and CDK10 increased the risk of CM (P-FDR < 0.05, beta < 0).

**Table 1:** Mendelian randomization results of drug-target gene and melanoma.

Exposure	Method	UK Biobank cohort (Discovery)					FINNGEN cohort (Replication)			
		SNPs	Beta	p-Value	OR (95% CI)	p-FDR	SNPs	Beta	p-Value	OR (95% CI)
CTSB	IVW	29	0.0012	2.36e-04	1.001(1.000-1.002)	0.0301	30	0.0708	0.0422	1.073(1.002-1.149)
CDK10	IVW	15	-0.0072	1.13e-04	0.992(0.989-0.996)	0.0249	18	-0.3937	0.0013	0.6745(0.5306-0.8573)
TPCN2	IVW	26	0.0011	2.26e-04	1.001(1.000-1.002)	0.0301	25	0.0799	0.039	1.083(1.004-1.168)
SLC9A1	IVW	4	0.0061	1.35e-04	1.006(1.003-1.009)	0.0272	4	-0.3408	0.03	0.7111(0.5227-0.9674)
RNASE6	IVW	36	-0.0012	1.67e-07	0.9987 (0.9982-0.9992)	0.0004	37	0.0918	5.47E-05	1.096(1.048-1.146)
LTA4H	IVW	35	0.0011	5.18e-05	1.001(1.000-1.002)	0.0171	37	0.0435	0.0381	1.044(1.002-1.088)
CD300LD	IVW	16	0.0013	6.96e-04	1.001(1.000-1.002)	0.0499	17	-0.1451	2.60E-05	0.8649(0.8084-0.9254)
ALDH3A2	IVW	5	0.0035	6.15e-04	1.003(1.001-1.005)	0.0487	6	0.2039	0.042	1.226(1.007-1.492)

IVW, inverse variance weighted; SNPs, single nucleotide polymorphisms.

The findings of the MR-PRESSO, horizontal pleiotropic, and heterogeneity tests are shown in **Table 2**. Due to the heterogeneity and pleiotropy levels of CDK10 and CM in the MR analysis (Q-P value < 0.05, P-Egger value < 0.05; **Table 2**), CDK10 was excluded. In addition, the beta values and odds ratios (ORs) of RNASE6, SLC9A1, and CD300LD were inconsistent between the two analyses (**Table 1**). Therefore, these three drug target genes were removed. Finally, the results of the MR analyses revealed that a total of four drug target genes have a causal relationship with melanoma. As shown in **Table 1**, as the expression levels of CTSB, TPCN2, LTA4H, and ALDH3A2 increased, the risk of CM increased (P-FDR < 0.05, OR > 1).

**Table 2:** Heterogeneity tests and horizontal pleiotropy of drug-target gene causally linked to melanoma in UK biobank.

Gene	Egger intercept	P-value^a^	Q-statistic (MR-Egger)	P-value^b^	MR-PRESSO P-value	P-value^c^
CTSB	-1.47E-4	0.398	30.42	0.295	9.91E-4	0.253
CDK10	-8.87E-4	0.258	138.47	4.25E-23	1.73E-3	<0.001
TPCN2	1.17E-4	0.347	28.92	0.222	1.09E-3	0.255
SLC9A1	9.55E-4	0.313	1.31	0.521	0.031	0.492
RNASE6	-9.76E-5	0.451	34.22	0.456	7.6E-06	0.459
LTA4H	1.18E-4	0.398	37.46	0.271	2.82E-4	0.285
CD300LD	-2.57E-4	0.111	12.84	0.538	4.02E-3	0.392
ALDH3A2	2.88E-4	0.579	1.85	0.603	0.01	0.804

^a^P-value: MR-Egger intercept P-value.

^b^P-value: Cochran’s Q test P-value.

^c^P-value: MR-PRESSO global p-value.

### 3.3 Screening of differentially expressed genes associated with CM

The GSE46517 dataset included skin samples from 31 CM patients and 7 healthy controls. After GEO2R screening for DEGs, 4,098 genes with P values less than 0.05 were screened, of which 2,025 were upregulated and 2,072 were downregulated (**Supplementary Table 5**). Compared with the four drug target genes identified above, the upregulated differentially expressed genes (DEGs) included LTA4H and ALDH3A2 (logFC > 0, P-adj < 0.05) (**Supplementary Table 5**), which agreed with our MR analysis findings. Because TPCN2 was not found among the DEGs, it was excluded. In addition, the results of the GEO2R analysis revealed that CTSB was expressed at low levels in CM cells (**Supplementary Table 5**), which was inconsistent with our MR results. Therefore, CTSB was also excluded.

### 3.4 Results of the PheWAS analysis

Using the PheWeb database, we conducted a PheWAS to identify the potential side effects of LTA4H and ALDH3A2. As shown in **Table 3**, the results of the PheWAS revealed significant causal relationships between LTA4H and skin cancer, supraventricular premature beats, nonmalignant ascites, and hypothermia. These results further confirm our findings that LTA4H can affect the incidence of skin cancer as well as the occurrence of other diseases. ALDH3A2 is associated with parathyroid diseases, pyelonephritis, ocular inflammation, fever of unknown origin, granulomatosis, and ulcerative colitis, but it is not directly associated with CM. Therefore, in the experimental section, we conducted cell experiments related only to LTA4H.

**Table 3:** PheWAS results of drug target genes based on the PheWeb database.

Gene	Disease	The highest p-value in the gene
LTA4H	skin cancer	1.8e-9
LTA4H	Supraventricular premature beat	4.2e-7
LTA4H	Ascites (non-malignant)	4.2e-7
LTA4H	Hypothermia/Chills	5.9e-7
ALDH3A2	Parathyroid disease	6.4e-8
ALDH3A2	Pyelonephritis	1.7e-7
ALDH3A2	Ocular inflammation	2.2e-7
ALDH3A2	Fever of unknown origin	5.1e-7
ALDH3A2	Granulomatosis	5.2e-7
ALDH3A2	Ulcerative colitis	8.0e-7

### 3.5 LTA4H knockdown in SK-MEL-28 melanoma cells reduced proliferation, invasion, migration, and mitochondrial membrane potential but increased apoptosis

**Figure 3:**
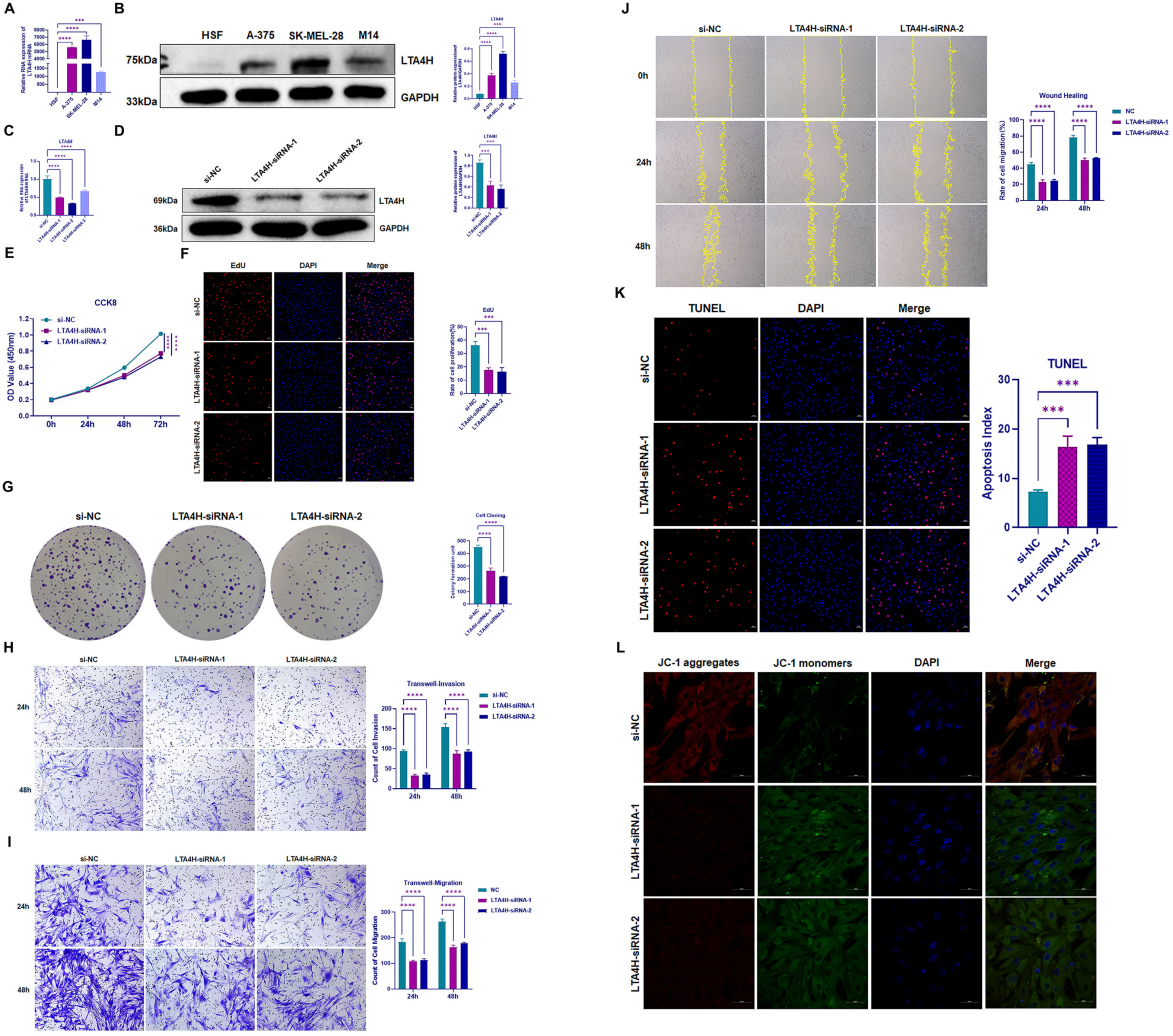
Expression of LTA4H in melanoma cell lines and the impact of LTA4H knockdown on cell viability, apoptosis, invasion, migration, and reactive oxygen species (ROS) in the SK-MEL-28 cell line. **(A)** RT-PCR analysis of LTA4H mRNA expression in HSF, A375, SK-MEL-28, and M14 cell lines showed significant differences compared to the A375, SK-MEL-28, and M14 cells (P<0.0001; P<0.0001; P<0.001, respectively). **(B)** Western blot analysis confirmed distinct protein expression levels of LTA4H in HSF, A375, SK-MEL-28, and M14 cell lines, with P<0.0001 for all comparisons. **(C)** RT-PCR analysis in SK-MEL-28 cells transfected with LTA4H-siRNA-1, -2, and -3 demonstrated significantly altered LTA4H mRNA expression levels, with P<0.0001 for all groups compared to the corresponding controls. **(D)** Western blotting of LTA4H protein expression in SK-MEL-28 cells transfected with LTA4H-siRNA-1 and -2 also showed significant changes, with P<0.001 for each siRNA compared to its respective control. **(E)** The CCK8 assay, measuring OD values at 450 nm, revealed significant reductions in cell viability in the LTA4H siRNA-1 and siRNA-2 groups compared to the si-NC group (P<0.0001 for both). **(F)** The EdU assay showed a notable decrease in the proportion of proliferating SK-MEL-28 cells in the LTA4H siRNA-1 and siRNA-2 groups (P<0.001 for both compared to si-NC). **(G)** The clonogenic formation assay indicated a significant reduction in the number of colonies formed by SK-MEL-28 cells in the LTA4H siRNA-1 and siRNA-2 groups (P<0.0001 for both). **(H)** Transwell invasion assays showed significantly fewer invasive cells in the LTA4H siRNA-1 and siRNA-2 groups at both 24 and 48 hours compared to the si-NC group (P<0.0001 for all). **(I)** Transwell migration assays similarly demonstrated significantly reduced migration of cells in the siRNA-1 and siRNA-2 groups at 24 and 48 hours compared to controls (P<0.0001 for all). **(J)** Wound healing assays revealed significantly decreased scratch healing areas in the siRNA-1 and siRNA-2 groups at 24 and 48 hours compared to the si-NC group (P<0.0001 for all). **(K)** TUNEL assays indicated a significant increase in apoptotic cells in the LTA4H siRNA-1 and siRNA-2 groups compared to the si-NC group (P<0.001 for both). **(L)** The JC-1 assay showed changes in mitochondrial membrane potential, visualized by shifts in JC-1 aggregates (red) to monomers (green), in the LTA4H siRNA-1 and siRNA-2 groups compared to the si-NC group.

First, we confirmed that LTA4H was expressed at both the mRNA and protein levels in melanoma cell lines such as A-375, SK-MEL-28, and M14, as well as in normal human dermal fibroblast HSF cells. RT-PCR and western blot analyses revealed that compared with HSF cells, A-375, SK-MEL-28, and M14 cells presented considerably greater LTA4H mRNA and protein expression. Notably, the SK-MEL-28 cell line presented the greatest expression (P < 0.0001), followed by A-375 (P < 0.0001), with M14 exhibiting relatively lower expression (P = 0.0001) (**Figure 3A, B**). To further analyses the role of LTA4H in melanoma, we generated three LTA4H siRNAs and confirmed their silencing efficacy in SK-MEL-28 cells. When RT-PCR was used to compare the mRNA levels of LTA4H between the LTA4H-siRNA-1, -siRNA-2, and -siRNA-3 groups and the si-NC group, all the differences were statistically significant, with a P value of less than 0.0001 (**Figure 3C**). Similarly, western blot assays revealed decreased LTA4H protein expression in the siRNA-1 (P = 0.0005) and siRNA-2 (P = 0.0003) groups (**Figure 3D**).

CCK-8 assays demonstrated that LTA4H knockdown in SK-MEL-28 cells led to significant changes in cell viability, as indicated by altered OD450 values at 24, 48, and 72 hours (**Figure 3E**). EdU assays further revealed a pronounced reduction in proliferation rates in the LTA4H-siRNA-1 (0.17 ± 0.01 vs. 0.36 ± 0.02, P = 0.0003) and LTA4H-siRNA-2 (0.16 ± 0.03 vs. 0.36 ± 0.02, P = 0.0002) groups compared with the control group (**Figure 3F**), indicating that LTA4H promotes SK-MEL-28 cell proliferation. Similarly, colony formation was significantly impaired upon LTA4H silencing (**Figure 3G**).

Transwell invasion and migration assays revealed that LTA4H knockdown substantially reduced the number of invasive and migratory cells at 24 and 48 hours (**Figures 3H, I**). These findings were corroborated by the results of the scratch wound assays, which revealed markedly shorter migration distances in the siRNA-treated groups (**Figure 3J**). Additionally, TUNEL assays revealed a significant increase in apoptosis in LTA4H-silenced cells (**Figure 3K**). Finally, JC-1 staining revealed a decrease in the mitochondrial membrane potential, characterized by decreased red fluorescence and increased green fluorescence in the knockdown groups (**Figure 3L**).

### 3.6 LTA4H overexpression in A-375 cells increased mitochondrial membrane potential, invasion, migration, and proliferation but decreased apoptosis

**Figure 4:**
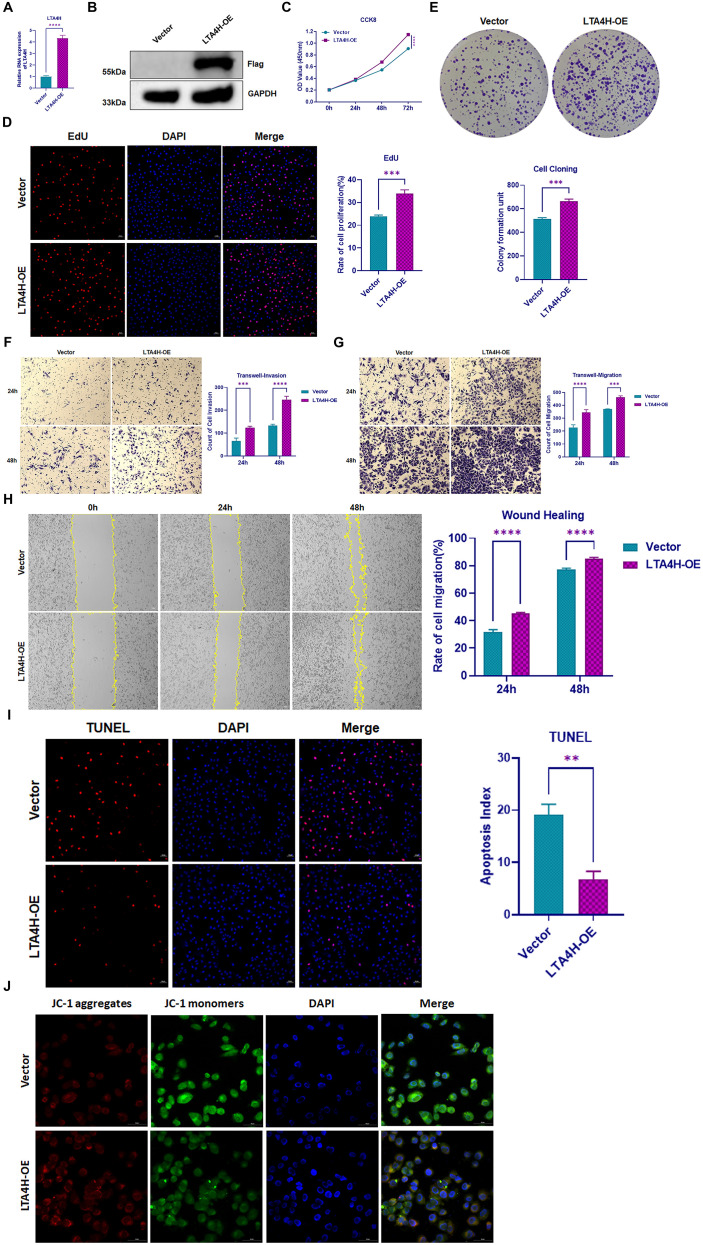
Impact of LTA4H overexpression (LTA4H-OE) on cell proliferation, apoptosis, invasion, migration, and mitochondrial membrane potential in A-375 cells. **(A)** RT-PCR analysis of LTA4H mRNA expression in LTA4H-OE group compared to Vector group. P<0.0001 vs LTA4H-OE group. **(B)** Western blot analysis of Flag-tagged LTA4H protein expression in LTA4H-OE group compared to Vector group. GAPDH was used as a loading control. P<0.0001 vs LTA4H-OE group. **(C)** CCK8 assay demonstrating the OD values at 450 nm of A-375 cells in Vector and LTA4H-OE groups at different time points (0, 24, 48, 72 hours). P<0.0001 vs LTA4H-OE group. **(D)** EdU assay revealing the proportion of proliferating A-375 cells in Vector and LTA4H-OE groups. P<0.001 vs LTA4H-OE group. **(E)** Clonogenic formation assay showing the number of colonies formed by A-375 cells in Vector and LTA4H-OE groups. P<0.001 vs LTA4H-OE group. **(F)** Transwell invasion assay demonstrating the number of invasive cells in Vector and LTA4H-OE groups at 24 and 48 hours. P<0.001 vs LTA4H-OE group at 24 hours; P<0.0001 vs LTA4H-OE group at 48 hours. **(G)** Transwell migration assay showing the number of migrated cells in Vector and LTA4H-OE groups at 24 and 48 hours. P<0.0001 vs LTA4H-OE group at 24 hours; P<0.001 vs LTA4H-OE group at 48 hours. **(H)** Wound healing assay revealing the scratch healing distances in Vector and LTA4H-OE groups at 24 and 48 hours. P<0.0001 vs LTA4H-OE group at 24 hours; P<0.0001 vs LTA4H-OE group at 48 hours. **(I)** TUNEL assay indicating the percentage of apoptotic A-375 cells in Vector and LTA4H-OE groups. P<0.01 vs LTA4H-OE group. **(J)** JC-1 assay revealing the mitochondrial membrane potential in Vector and LTA4H-OE groups.

**Figure 5:**
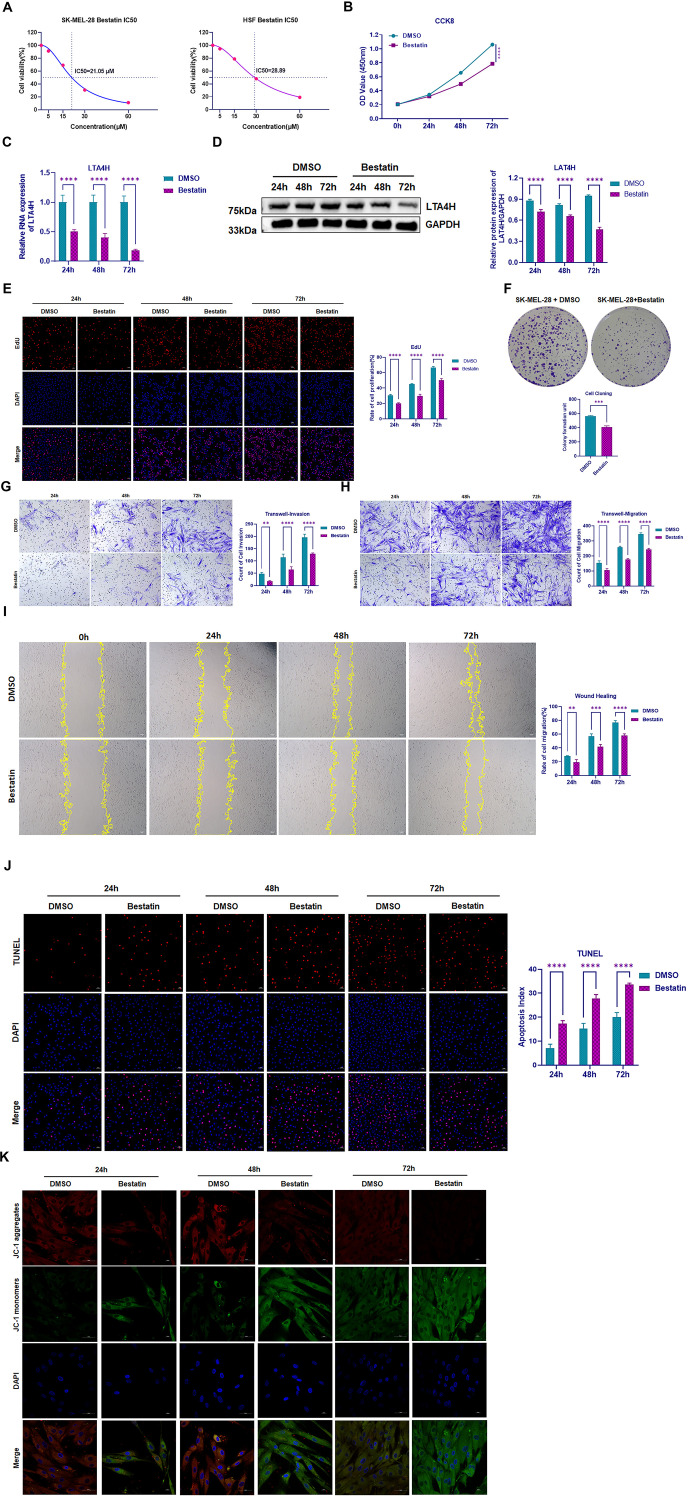
Impact of Bestatin on the proliferation, migration, invasion, apoptosis, mitochondrial membrane potential, and LTA4H expression in SK-MEL-28 cells. **(A)** The dose-response curve illustrates the impact of Bestatin on the viability of SK-MEL-28 cells and HSF cells. The IC50 value of Bestatin for SK-MEL-28 is determined to be 21.05 μM, while for HSF cells, it is ascertained at 28.89 μM. **(B)** The CCK8 assay was employed to measure the optical density at 450nm (OD450) of DMSO-treated and Bestatin-treated groups over a period of 24 hours, 48 hours, and 72 hours. **(C)** RT-PCR analysis of LTA4H mRNA expression in SK-MEL-28 cells treated with DMSO or Bestatin at different time points (24, 48, 72 hours). **(D)** Western blot analysis of LTA4H protein expression in SK-MEL-28 cells treated with DMSO or Bestatin at different time points (24, 48, 72 hours). GAPDH was used as a loading control. **(E)** EdU assay showing the cell viability of SK-MEL-28 cells treated with DMSO or Bestatin at different time points (24, 48, 72 hours). P<0.0001 vs Bestatin group at 24 hours; P<0.0001 vs Bestatin group at 48 hours; P<0.0001 vs Bestatin group at 72 hours. **(F)** Clonogenic formation assay revealing the number of colonies formed by SK-MEL-28 cells treated with DMSO or Bestatin. P<0.001 vs Bestatin group. **(G)** Transwell invasion assay demonstrating the number of invasive cells in SK-MEL-28 cells treated with DMSO or Bestatin at different time points (24, 48, 72 hours). P<0.01 vs Bestatin group at 24 hours; P<0.0001 vs Bestatin group at 48 hours; P<0.0001 vs Bestatin group at 72 hours. **(H)** Transwell migration assay revealing the number of migratory cells in SK-MEL-28 cells treated with DMSO or Bestatin at different time points (24, 48, 72 hours). P<0.0001 vs Bestatin group at 24 hours; P<0.0001 vs Bestatin group at 48 hours; P<0.0001 vs Bestatin group at 72 hours. **(I)** Wound healing assay showing the percentage of scratch healing areas in SK-MEL-28 cells treated with DMSO or Bestatin at different time points (24, 48, 72 hours). P<0.01 vs Bestatin group at 24 hours; P<0.001 vs Bestatin group at 48 hours; P<0.0001 vs Bestatin group at 72 hours. **(J)** TUNEL assay indicating the apoptosis in SK-MEL-28 cells treated with DMSO or Bestatin at different time points (24, 48, 72 hours). P<0.0001 vs Bestatin group at 24 hours; P<0.0001 vs Bestatin group at 48 hours; P<0.0001 vs Bestatin group at 72 hours. **(K)** JC-1 assay revealing the mitochondrial membrane potential in SK-MEL-28 cells treated with DMSO or Bestatin at different time points (24, 48, 72 hours).

To determine how LTA4H affects melanoma, we designed an LTA4H overexpression plasmid and evaluated its effects on A-375 cells. Consequently, PCR analysis demonstrated that the LTA4H-overexpressing cells had a significantly higher mRNA content than the control cells (4.32 ± 0.27 vs. 1.00 ± 0.09, P < 0.0001; **Figure 4A**). These findings were further confirmed at the protein level using western blotting, as depicted in **Figure 4B**. CCK8 assays also revealed that these cells proliferated faster in the long term, as indicated by higher OD values (**Figure 4C**). The results also revealed increased Ki-67 expression in the LTA4H-OE group (0.34 ± 0.02 vs. 0.24 ± 0.00, P = 0.0005; **Figure 4E**). The number of colonies formed was greater in the LTA4H-OE group (663 ± 19 compared with 514 ± 10, P = 0.0005; **Figure 4E**). The proliferation and migratory capacity of these cells were also measured and found to be greater than those of the control cells via Transwell assays (**Figures 4F** and **5G**). Scratch tests confirmed these findings, as the LTA4H-overexpressing cells migrated farther than the control cells did after the culture period (**Figure 4H**). TUNEL analysis revealed that the number of apoptotic cells was lower than that in the control group (0.07 ± 0.02 vs. 0.19 ± 0.02; P = 0.0010), as presented in **Figure 4I**. Finally, JC-1 staining revealed an increased mitochondrial membrane potential in the LTA4H-OE group, as indicated by increased red fluorescence and reduced green fluorescence (**Figure 4J**).

### 3.7 Bestatin treatment inhibited the growth, invasion, and migration of SK-MEL-28 cells; reduced the mitochondrial membrane potential; and increased cell death

A concentration gradient of 10–100 μM bestatin was used to assess its cytotoxicity in SK-MEL-28 and HSF cells using a CCK-8 assay. The IC_50_ values were 21.05 μM for SK-MEL-28 (95% CI: 19.2–22.9) and 28.89 μM for HSF (95% CI: 26.1–31.7), demonstrating selective inhibition against melanoma cells (**Figure 5A**).

The results of time-course experiments revealed a significant decrease in the OD450 of bestatin-treated cells compared with that of the DMSO control at 24, 48, and 72 h (P < 0.0001; **Figure 5B**). RT-qPCR analysis revealed that LTA4H mRNA expression gradually decreased over time in the bestatin group compared with that in the DMSO group (**Figure 5C**). Similarly, western blot analysis revealed progressively lower LTA4H protein levels in bestatin-treated cells (**Figure 5D**).

EdU assays revealed a marked reduction in cell proliferation at 24, 48, and 72 h after bestatin treatment (**Figure 5E**). Colony formation was also significantly suppressed in the bestatin group (**Figure 5F**). Transwell invasion assays revealed fewer invading cells in the bestatin group at all time points (**Figure 5G**), and migration assays similarly demonstrated decreased cell migration at 24 and 48 h (**Figure 5H**). Scratch-wound healing assays further confirmed the impaired migration of bestatin-treated cells (**Figure 5I**).

TUNEL staining revealed increased apoptosis in the bestatin group compared with the DMSO control group at 24, 48, and 72 h (**Figure 5J**). JC-1 immunofluorescence staining revealed decreased red fluorescence and enhanced green fluorescence in bestatin-treated cells, reflecting a decrease in the mitochondrial membrane potential (**Figure 5K**).

### 3.8 Bestatin targets LTA4H to efficiently prevent A-375 cells from proliferating, invading, and migrating while concurrently promoting apoptosis

**Figure 6:**
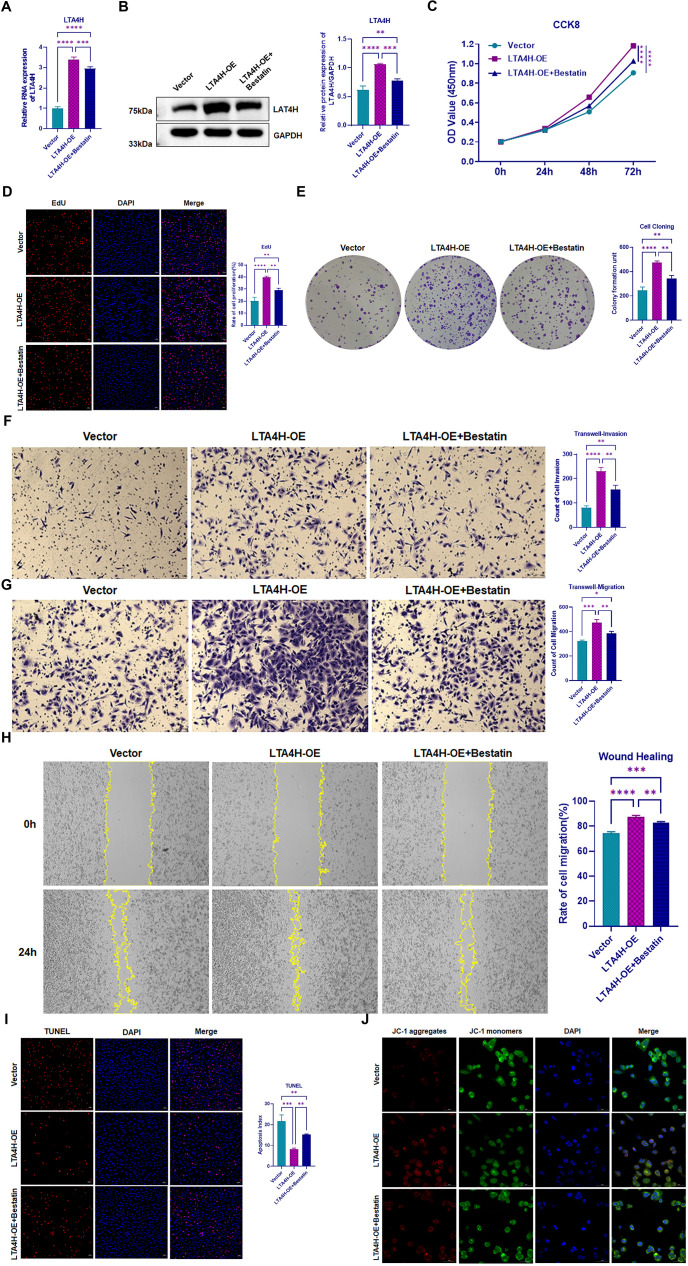
Impact of LTA4H overexpression (LTA4H-OE) and Bestatin on cell proliferation, apoptosis, invasion, migration, and mitochondrial membrane potential in A-375 cells. **(A)** RT-PCR analysis of LTA4H mRNA expression in Vector, LTA4H-OE, and LTA4H-OE+Bestatin groups. P<0.0001 vs LTA4H-OE group; P<0.0001 vs LTA4H-OE+Bestatin group. **(B)** Western blot analysis of LTA4H protein expression in Vector, LTA4H-OE, and LTA4H-OE+Bestatin groups. GAPDH was used as a loading control. P<0.0001 vs LTA4H-OE group; P<0.001 vs LTA4H-OE+Bestatin group. **(C)** CCK8 assay showing the OD values at 450 nm of A-375 cells in Vector, LTA4H-OE, and LTA4H-OE+Bestatin groups at different time points (0, 24, 48, 72 hours). P<0.0001 vs LTA4H-OE group; P<0.0001 vs LTA4H-OE+Bestatin group. **(D)** EdU assay revealing the proportion of proliferating A-375 cells in Vector, LTA4H-OE, and LTA4H-OE+Bestatin groups. P<0.001 vs LTA4H-OE group; P<0.01 vs LTA4H-OE+Bestatin group. **(E)** Clonogenic formation assay showing the number of colonies formed by A-375 cells in Vector, LTA4H-OE, and LTA4H-OE+Bestatin groups. P<0.0001 vs LTA4H-OE group; P<0.01 vs LTA4H-OE+Bestatin group. **(F)** Transwell invasion assay demonstrating the number of invasive A-375 cells in Vector, LTA4H-OE, and LTA4H-OE+Bestatin groups at different time points (24, 48 hours). P<0.0001 vs LTA4H-OE group at 24 hours; P<0.01 vs LTA4H-OE+Bestatin group at 48 hours. **(G)** Transwell migration assay showing the number of migratory A-375 cells in Vector, LTA4H-OE, and LTA4H-OE+Bestatin groups at different time points (24, 48 hours). P<0.001 vs LTA4H-OE group; P<0.01 vs LTA4H-OE+Bestatin group at 48 hours. **(H)** Scratch assays evaluated the migration distances of A-375 cells in the Vector, LTA4H-OE, and LTA4H-OE+Bestatin groups at 24 and 48 hours. P<0.0001 compared to LTA4H-OE group; P<0.01 at 48 hours. **(I)** TUNEL assays determined the apoptosis rates of A-375 cells in the Vector, LTA4H-OE, and LTA4H-OE+Bestatin groups. P<0.001 compared to LTA4H-OE group; P<0.01 at 48 hours. **(J)** JC-1 assays measured the mitochondrial membrane potential of A-375 cells in the Vector, LTA4H-OE, and LTA4H-OE+Bestatin groups.

To further validate the role of bestatin in modulating LTA4H activity in melanoma, RT-PCR was used to quantify LTA4H mRNA levels. The results revealed that LTA4H mRNA expression in the LTA4H-OE group was significantly greater than that in the vector control group (3.40 ± 0.12 vs. 1.00 ± 0.08, P < 0.0001). Importantly, bestatin treatment markedly reduced LTA4H expression in the LTA4H-OE group (2.95 ± 0.09 vs. 3.40 ± 0.12, P = 0.0003) (**Figure 6A**). Western blot analysis further confirmed that LTA4H protein levels were substantially elevated in the LTA4H-OE group compared with those in the vector control group (1.06 ± 0.01 vs. 0.61 ± 0.07, P < 0.0001), and this increase was significantly suppressed by bestatin (0.78 ± 0.03 vs. 1.06 ± 0.01, P = 0.0006) (**Figure 6B**).

CCK-8 assays revealed that cell viability, as reflected by the OD450 values at 24 h, 48 h, and 72 h, was significantly greater in the LTA4H-OE group than in the vector control group. In contrast, bestatin treatment clearly reduced the OD values of LTA4H-OE cells at all time points, indicating decreased cell viability (**Figure 6C**). EdU assays revealed a substantially increased proliferation rate in the LTA4H-OE group compared with that in the vector control group (0.40 ± 0.01 vs. 0.20 ± 0.03, P < 0.0001), which was attenuated by bestatin (0.29 ± 0.02 vs. 0.40 ± 0.01, P = 0.0016) (**Figure 6D**). Colony formation assays likewise revealed a pronounced increase in colony number in the LTA4H-OE group compared with that in the vector control group (472 ± 14 vs. 246 ± 26, P < 0.0001), and this effect was significantly reversed by bestatin (343 ± 24 vs. 472 ± 14, P = 0.0010) (**Figure 6E**).

Transwell invasion assays revealed that the number of invading cells was significantly greater in the LTA4H-OE group than in the control group at both 24 h and 48 h, and this increase was suppressed by bestatin (**Figure 6F**). Similarly, Transwell migration assays revealed enhanced migratory activity in the LTA4H-OE group, which was markedly reduced upon bestatin treatment (**Figure 6G**). Scratch-wound assays further supported these results: the migration distance was significantly greater in the LTA4H-OE group than in the vector control group (0.87 ± 0.01 vs. 0.75 ± 0.01, P < 0.0001), and compared with LTA4H alone, bestatin shortened the migration distance (0.83 ± 0.01 vs. 0.87 ± 0.01, P = 0.0045) (**Figure 6H**).

TUNEL apoptosis assays confirmed that bestatin promotes apoptosis in A-375 cells via LTA4H inhibition. The percentage of apoptotic cells was significantly lower in the LTA4H-OE group than in the vector control group (0.08 ± 0.00 vs. 0.22 ± 0.03, P = 0.0002), whereas it was substantially greater in the LTA4H-OE + bestatin group than in the LTA4H-OE group (0.15 ± 0.00 vs. 0.08 ± 0.00, P = 0.0068) (**Figure 6I**). JC-1 immunofluorescence staining revealed that the mitochondrial membrane potential, indicated by increased red fluorescence and decreased green fluorescence, was elevated in the LTA4H-OE group. In contrast, the LTA4H-OE + bestatin group displayed increased green fluorescence and decreased red fluorescence, reflecting a reduction in mitochondrial membrane potential (**Figure 6J**).

## 4 Discussion

To identify drug targets for cutaneous melanoma (CM), we integrated GWAS, pharmacogenomic, and eQTL data in a Mendelian randomization (MR) study. The results linked higher LTA4H expression to an increased risk of CM. Compared with normal fibroblasts, melanoma cells had elevated LTA4H levels. LTA4H knockdown in SK-MEL-28 cells reduced invasion, migration, proliferation, EMT, and cell-cycle protein expression but increased apoptosis and decreased the mitochondrial membrane potential. Conversely, LTA4H overexpression in A-375 cells increased proliferation, invasion, migration, EMT, and the expression of cell-cycle proteins but decreased apoptosis and increased the mitochondrial membrane potential. Bestatin treatment suppressed proliferation, migration, and invasion and increased apoptosis while reducing the mitochondrial membrane potential in SK-MEL-28 cells. In A-375 cells, bestatin inhibited proliferation, invasion, and migration and promoted apoptosis through LTA4H inhibition. Collectively, these findings indicate that LTA4H is highly expressed in CM cells and drives malignant phenotypes, whereas bestatin counteracts these effects by downregulating LTA4H expression.

Leukotriene A4 hydrolase (LTA4H) is an inflammatory mediator that has garnered attention because of its role in the development of chronic lung diseases and various cancers (23). Its main function involves facilitating the biochemical transformation of leukotriene A4 (LTA4), a highly active and unstable intermediate, into leukotriene B4 (LTB4), a more stable and biologically active compound. This transformation is crucial because LTB4 performs important biological functions, including chemotaxis (the process by which cells move in response to chemical signals), endothelial adhesion, and leukocyte activation, all of which are essential for immune responses (24–26). LTA4H is in fact overexpressed in human skin cancer tissues, according to significant research by Oi and colleagues (27). This overexpression indicates that LTA4H has a potential role in the progression and development of cancer. To further study the significance of this overexpression, they conducted experiments in a mouse model of skin cancer. When the LTA4H gene was knocked out in these mice, the incidence of skin cancer significantly decreased. In an *in vivo* skin cancer mouse model, LTA4H deletion dramatically decreased the development of skin cancer, and LTA4H depletion resulted in cell cycle arrest at the G0/G1 phase. These results reveal a novel way in which LTA4H contributes to the development of cancer.

The dipeptide immunomodulator bestatin (ubenimex) was identified in Streptomyces olivoreticuli culture supernatant (28) and is a well-established LTA4H inhibitor (29, 30). Zhao et al. reported that LTA4H and BLT1, which are highly expressed in colitis, are associated with colon cancer (31). The survival probability of people with colorectal cancer is also negatively correlated with high expression of LTA4H and BLT1 (31). Additionally, bestatin strongly inhibited tumour cell growth in individuals with colorectal cancer by suppressing the expression of the Ki-67 protein, which markedly reduced the proliferation of tumour cells (31). The growth and colony structure of colorectal cancer cells were markedly reduced by LTA4H knockdown or bestatin therapy. By blocking LTA4H, bestatin can prevent melanoma cells from proliferating, invading, and migrating, and it can also increase the expression of proteins linked to death in melanoma cells.

Our study has numerous noteworthy advantages. First, this research is the first to apply MR to identify drug targets associated with CM, and it utilizes the most extensive publicly available GWAS data on CM risk, which significantly enhances the innovation and reliability of this research. Moreover, the MR findings were validated in two important cohorts, a validation process that strengthens the reliability of the results, greatly reduces the likelihood of false positives, and potentially improves the effectiveness of clinical trials. This study is also the first to validate the high expression of LTA4H in melanoma cells across three melanoma cell lines—SK-MEL-28, A-375, and M14—indicating that our MR research findings are highly reliable. Second, our study is the first to verify that bestatin can inhibit the malignant functions of melanoma cells by suppressing LTA4H expression, providing new insights for the treatment of melanoma. This study has several significant drawbacks. First, the experiments in this study were conducted only in melanoma cells and were not validated in animal experiments. Second, we have not yet explored the upstream and downstream mechanisms of LTA4H overexpression in melanoma cells to elucidate the pathways through which LTA4H affects melanoma, and further *in vitro* and *in vivo* research is needed. Third, the CM data derived from FinnGen R11 are less extensive than those in the most recent FinnGen R13 release because of the shorter follow-up and fewer accrued cases; this limitation may have influenced the results of our study. Finally, it should be noted that the list of druggable genes used in this study was derived from an earlier release, which may not fully reflect the current landscape of the druggable genome. Given the rapid expansion of recognized druggable targets in recent years, our analysis likely did not capture all potential candidate genes, particularly those newly identified or belonging to emerging target classes. This limitation may have influenced target prioritization and should be considered when our findings are interpreted. Future work incorporating more up-to-date and comprehensive resources is needed to validate these results.

## 5 Conclusion

In this study, through MR analysis, we revealed a strong causal relationship between LTA4H and CM at the genetic level. We verified that LTA4H is highly expressed in three CM cell lines. Moreover, high expression of LTA4H leads to increased proliferation, invasion, and migration in melanoma cells, along with decreased apoptosis. Additionally, bestatin can inhibit the proliferation, invasion, and migration of melanoma cells by suppressing the expression of LTA4H and promoting the expression of apoptosis-related proteins. Our findings provide a new perspective for the treatment of CM.

## Data Availability

The original contributions presented in the study are included in the article/**Supplementary Material**. Further inquiries can be directed to the corresponding authors.
